# Left Atrial Function in Young Patients With Cryptogenic Stroke and Patent Foramen Ovale: A Left Atrial Longitudinal Strain Study

**DOI:** 10.3389/fneur.2020.536612

**Published:** 2020-11-05

**Authors:** Julie Gazagnes, Cédric Gollion, Pauline Fournier, Eve Cariou, Vincent Larrue, Olivier Lairez

**Affiliations:** ^1^Department of Neurology, University Hospital, Toulouse, France; ^2^Medical School, Toulouse III Paul Sabatier University, Toulouse, France; ^3^Department of Cardiology, University Hospital, Toulouse, France; ^4^Cardiac Imaging Center, Toulouse University Hospital, Toulouse, France; ^5^Department of Nuclear Medicine, Toulouse University Hospital, Toulouse, France

**Keywords:** cryptogenic stroke, stroke in the young, patent foramen ovale, left atrial function, speckle tracking

## Abstract

**Background:** The study of left atrial (LA) longitudinal strain by speckle tracking is a reliable method for analyzing LA function that could provide relevant information in young patients with cryptogenic stroke (CS). The aim of this study was to investigate whether the presence of a patent foramen ovale (PFO) impacts the LA longitudinal strain in a population of young patients with first CS.

**Methods and Results:** Patients aged 18 to 54 years, treated consecutively in a university hospital for first CS, were included in this study. The presence of a PFO and an atrial septal aneurysm (ASA) was investigated using transesophageal echocardiography and transcranial Doppler. Speckle tracking analysis was performed on transthoracic echocardiography, allowing the measurement of global, passive, and active longitudinal LA strain, corresponding to the reservoir, conduit, and contractile function, respectively. A total of 51 patients were included in the study. In a multivariable analysis, overweight was associated with reduced global and passive LA longitudinal strain (*P* = 0.013 and *P* = 0.018, respectively), and hypertension was associated with reduced active LA longitudinal strain (*P* = 0.049). LA longitudinal strain was not different between patients with PFO or PFO plus ASA and patients without PFO.

**Conclusion:** LA longitudinal strain in young subjects with CS was impaired in the presence of overweight and hypertension, but not of PFO or PFO plus ASA.

## Introduction

Stroke is far more common in the elderly than in the young. However, recent epidemiological studies have shown that the incidence of stroke is increasing in young subjects (1). The etiological spectrum of stroke in the young is different from those of older subjects. Moreover, between 30 and 50% of strokes in the young are classified as cryptogenic stroke (CS) despite extensive etiological workup (2, 3). Impaired left atrial (LA) mechanical function is one of the possible causes of seemingly CS (4). Impaired LA function may be a factor of blood stasis and thrombus formation (5). A large population-based cohort study showed that impaired LA function was associated withi incident cerebrovascular events independent of known cerebrovascular risk factors and incident atrial fibrillation (6). Impaired LA function has been associated with age, hypertension, obesity, and diabetes mellitus (7–9). In addition, a few studies have suggested that impaired LA function may be associated with patent foramen ovale (PFO) or atrial septal aneurysm (ASA) in stroke patients (10, 11).

Speckle tracking is an ultrasound technique based on tracking the displacement of acoustic markers during the cardiac cycle, reflecting the myocardial deformation. This non-invasive technique has been validated for the assessment of LA function, allowing the measurement of global, passive, and active longitudinal strain, reflecting LA reservoir (storage of blood during left ventricular systole), conduit (passage of blood from the pulmonary veins to the left ventricle during early diastole), and contractile (filling of left ventricle through LA active contraction during end of diastole) functions, respectively (12, 13).

In this study, using speckle tracking analysis in young patients with first CS, we sought to determine whether PFO or PFO plus ASA were associated with LA function impairment.

## Methods

### Study Population

Consecutive patients aged 18 to 54 years, treated for first-ever CS in a tertiary hospital, were included in this retrospective study. Patients with cerebral venous thrombosis, subarachnoid hemorrhage with secondary brain ischemia, or transient ischemic attack as defined by transient neurologic dysfunction without evidence of infarction on brain imaging were not included. Clinical, biological, and radiological data from all individual patients were reviewed using the electronic database. Hypertension, diabetes, and migraine were diagnosed by history. Overweight (including obesity) was defined as body mass index (BMI) >24.9. Hypertension was defined as persistent systolic blood pressure >130 mmHg or diastolic blood pressure >80 mmHg, as documented before stroke or treatment with antihypertensive drugs before stroke. Diabetes was defined as a previous diagnosis of type 1 or type 2 diabetes. Tobacco use was recorded in patients who were currently smoking. Hyperlipidemia was defined as elevated low-density lipoprotein cholesterol >1.6 g/L or hypertriglyceridemia >2.0 g/L.

The study conformed to the principles outlined in the Declaration of Helsinki. All patients were informed that clinical data collected during their hospitalization could be used for research purposes and gave their consent. The study was approved by our Institutional Review Board (internal reference RnlPH 2019-73).

### Stroke Diagnosis

Stroke was diagnosed according to current recommendations as an episode of acute neurological deficit corresponding with an acute ischemic lesion on brain magnetic resonance imaging (MRI). CS was retained after a negative complete diagnostic workup including brain MRI, ECG, 72-h telemetry, routine blood tests, and non-invasive angiography of cerebral and cervical vessels using MRI or computed tomography angiography, carotid duplex ultrasonography, and, in patients without a definite cause of stroke after an initial evaluation, transthoracic (TTE) and transesophageal (TEE) echocardiography. Additional investigations including 24-h Holter monitoring, cerebrospinal fluid analysis, and testing for thrombophilia were performed in selected patients with suggestive findings on initial evaluation or without a potential cause of stroke after completion of echocardiography.

The etiology of stroke was classified according to the ASCOD classification system (A, atherosclerosis; S, small-vessel disease; C, cardiac pathology; O, other causes; D, dissection). This classification system assigns a degree of likelihood of causal relationship to every potential disease (1 for potentially causal, 2 for causality is uncertain, 3 for unlikely causal but the disease is present, 0 for absence of a disease, and 9 for insufficient workup to rule out the disease) (15).

CS was diagnosed in patients without an ASCOD grade 1 cause of stroke. For the purpose of this study and in accordance with the ASCOD classification, patients with PFO as the only potential cause of stroke were classified as CS.

### Echocardiography

TTE and TEE were performed with a commercially available ultrasound Vivid E95 system (GE Vingmed Ultrasound AS, Horten, Norway) using either a 2.5-MHz transthoracic transducer or an 8-MHz transesophageal transducer, allowing a full-fledged analysis of archived sequences.

The presence of PFO and ASA were assessed by TEE with a contrast study performed at rest and during provocative maneuvers (Valsalva and cough test) according to guidelines (16). The contrast study was considered positive if ≥3 microbubbles appeared in the left atrium, either spontaneously or after provocative maneuvers, within three cardiac cycles after complete opacification of the right atrium (17). The degree of shunting was defined as small (grade 1; <20 bubbles) or large (grade 2; ≥20 bubbles). In case of negative TEE, PFO was to be diagnosed in the presence of a right-to-left shunt on transcranial Doppler, after eliminating other causes of right-to-left shunt. ASA was defined as excursion of the septal tissue of >10 mm from the plane of the atrial septum into the right atria or LA or a combined total excursion to the right and to the left of 15 mm (12).

Left ventricular (LV) ejection fraction was measured using the modified biplane Simpson's rule. Peak early (E) and late (A) waves were derived from pulse wave Doppler of mitral inflow.

LA volumes were measured in the apical four- and two-chamber views. The most suitable cardiac cycle was chosen for each view. The assessed parameters in each view included LA maximal volume (*V*_max_), at mitral valve opening, minimal volume (*V*_min_), at mitral valve closure, and pre-LA contraction volume (*V*_preA_) at the onset of the P wave. The volumetric parameters of the LA function were calculated as follows (9, 18): total emptying volume (ml) = *V*_max_-*V*_min_, passive emptying volume (ml) = *V*_max_-*V*_preA_, active emptying volume (ml) = *V*_preA_-*V*_min_, total emptying fraction (%) = total emptying volume/*V*_max_ × 100, passive emptying fraction (%) = passive emptying volume/*V*_max_ × 100, and active emptying fraction (%) = active emptying volume/*V*_preA_ × 100.

**Figure 1:**
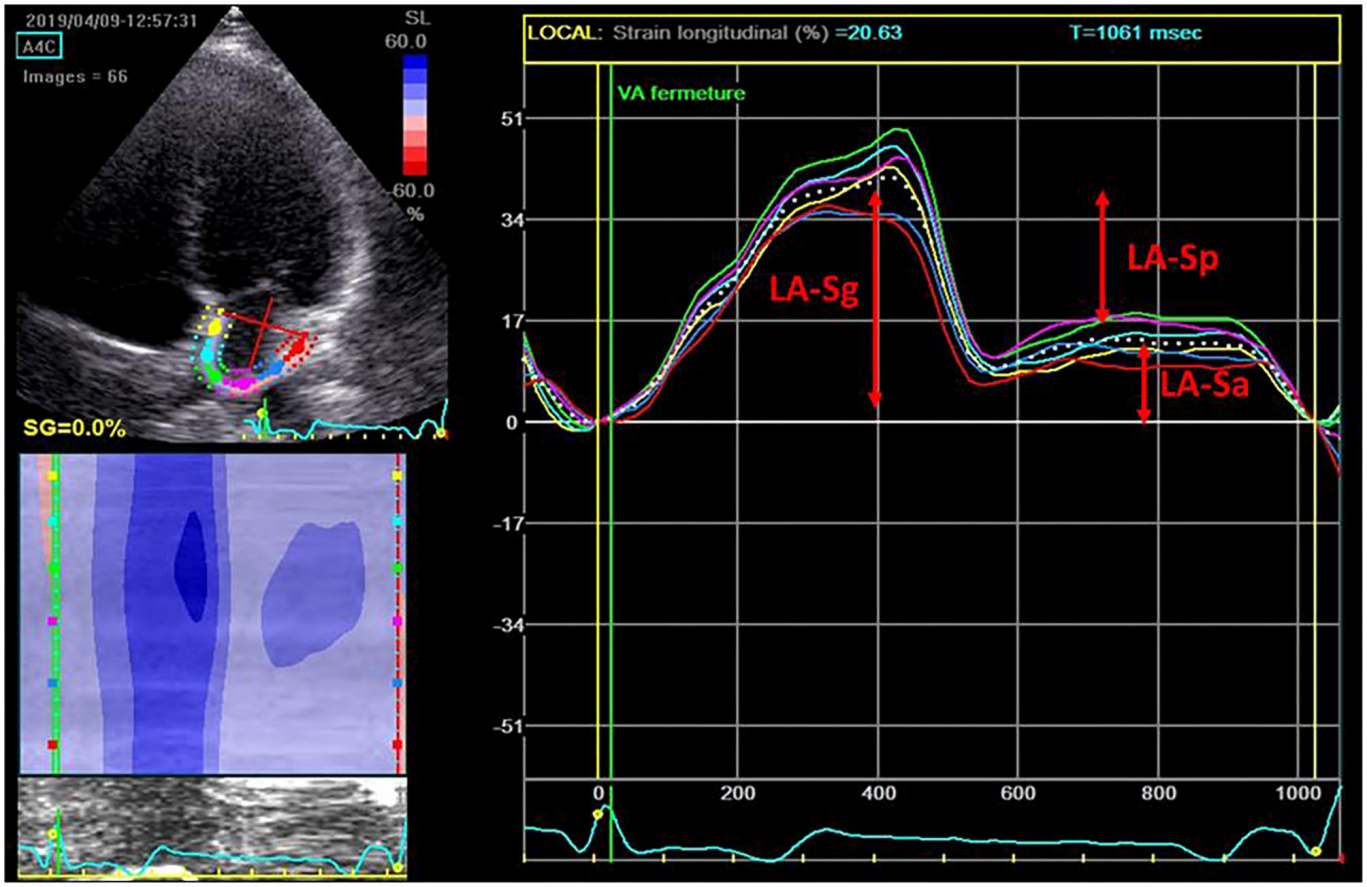
Strain curve obtained after manual tracing of the left atrial (LA) border. LA-Sa, LA longitudinal active strain; LA-Sg, LA longitudinal global strain; LA-Sp, LA longitudinal passive strain.

For speckle tracking analysis, the frame rate was set between 60 and 80 frames per second. The reference point was set at the beginning of the QRS complex. LA endocardial surface was manually traced in both four- and two-chamber views by a point-and-click approach (19). An epicardial surface tracing was then automatically generated by the system, thus creating a region of interest (13). The accuracy of tracking was visually confirmed throughout the cardiac cycle and confirmed from the morphology of the strain curves. If necessary, manual correction could be made or, if still non-acceptable, the segment was excluded from the analysis. Strain curves were generated for each segment. LA global longitudinal strain, active longitudinal strain, and passive longitudinal strain were measured by averaging the values observed in all available LA segments (12 when all four- and two-chamber segments were suitable), as shown in Figure 1.

### Statistical Analysis

Continuous variables were expressed as mean ± standard deviation or median and interquartile range. Intra- and inter-rater reliability for LA strain measurement were assessed by intra-class correlation coefficient from 10 randomly selected patients reanalyzed by two observers. Nominal values were expressed as numbers and percentages. We used Mann–Whitney rank sum test and Fisher exact test for comparison of continuous and nominal variables, respectively. A multivariable analysis was performed using logistic regression analysis. Reduced LA strain was defined as LA strain < median value. All tests were bilateral. Differences were considered as statistically significant for a *P* < 0.05. All analyses were performed using SPSS, version 20 (SPSS Inc., Chicago, IL, United States).

## Results

Fifty-one patients were included in this study. The mean age was 42 ± 9 years; 34 (67%) patients were male. The patients' characteristics are summarized in Table 1.

**Table 1:** Patient characteristics (values are numbers with percentages in parentheses unless otherwise indicated).

**Age mean ± SD, years**	**42 ± 9**
Men	34 (67)
Hypertension	12 (24)
Overweight	25 (49)
Smoking	28 (55)
Dyslipidemia	6 (12)
Diabetes	4 (8)
PFO	21 (41)
PFO-ASA	12 (24)
Spontaneous R–L shunt	18 (35)
Large shunt (grade 2)	12 (24)

Intra- and inter-rater reliability for LA global longitudinal strain were good or excellent with intra-class correlation coefficient (95% CI) of 0.903 (0.569–0.978; *P* = 0.002) and 0.889 (0.509–0.975; *P* = 0.003), respectively.

The associations of traditional cardiovascular risk factors with LA longitudinal strain in univariate analysis are summarized in Table 2. LA global longitudinal strain was reduced in older patients (*P* = 0.009) and in the presence of hypertension (*P* = 0.002), diabetes (*P* = 0.049), and overweight (*P* = 0.001). Older age (*P* < 0.001), overweight (*P* = 0.004), and non-smoking (*P* = 0.034) were associated with reduced LA passive longitudinal strain. LA active strain was not associated with any of the variables tested.

**Table 2:** Left atrial (LA) longitudinal strain values according to age, sex, and cardiovascular risk factors (values are mean ± SD).

		**LA global strain**	***P***	**LA active strain**	***P***	**LA passive strain**	***P***
Age, years^a^	≥45 (*N* = 22)	35.0 ± 8.4	0.009	16.5 ± 5.4	0.506	17.2 ± 5.1	<0.001
	<45 (*N* = 29)	41.2 ± 7.7		16.9 ± 4.4		24.7 ± 7.2	
Male sex	Yes (*N* = 17)	37.6 ± 8.3	0.281	17.2 ± 5.2	0.549	20.7 ± 7.6	0.134
	No (*N* = 34)	40.4 ± 8.8		15.9 ± 4.0		23.3 ± 6.5	
Overweight	Yes (*N* = 25)	34.5 ± 8.4	0.001	15.9 ± 4.4	0.250	18.7 ± 7.3	0.004
	No (*N* = 26)	42.5 ± 6.5		17.5 ± 5.1		24.3 ± 6.3	
Smoking	Yes (*N* = 21)	40.6 ± 6.9	0.057	16.1 ± 4.1	0.592	24.3 ± 7.5	0.034
	No (*N* = 30)	37.1 ± 9.3		17.2 ± 5.3		19.7 ± 6.6	
Hypertension	Yes (*N* = 12)	31.9 ± 7.0	0.002	14.7 ± 4.0	0.065	18.4 ± 6.1	0.083
	No (*N* = 39)	40.6 ± 7.9		17.4 ± 4.9		22.6 ± 7.4	
Diabetes	Yes (*N* = 4)	30.6 ± 6.4	0.049	14.5 ± 4.9	0.405	16.0 ± 1.9	0.083
	No (*N* = 47)	39.2 ± 8.3		16.9 ± 4.8		22.1 ± 7.4	
Hyperlipidemia	Yes (*N* = 6)	34.8 ± 7.8	0.188	18.6 ± 6.1	0.366	16.4 ± 3.4	0.058
	No (*N* = 45)	39.0 ± 8.5		16.5 ± 4.6		22.3 ± 7.4	

a *Age was dichotomized according to median value*.

Twenty-one (41%) patients had PFO, including four patients with negative TEE but with a right-to-left shunt on transcranial Doppler. Ten (19.6%) PFOs were associated with an ASA. The patients with PFO were younger (38.8 ± 10.7 vs. 43.8 ± 6.8 years, *P* = 0.101) and had less hypertension (14.3 vs. 30%; *P* = 0.315) and diabetes (4.8 vs. 10%; *P* = 0.634) than the patients without PFO. However, none of these differences were statistically significant. BMI was similar in both groups (25.9 vs. 26.1; *P* = 0.716).

PFO and PFO plus ASA were not associated with any modification of global, active, or passive longitudinal LA strain. Large right-to-left shunt, defined as more than 20 bubbles on TEE and present in 12 patients (24%), was not associated with altered LA strain (Table 3). Among conventional LA function parameters, only LA total emptying volume was increased in patients with PFO (*P* = 0.025, Supplementary File).

**Table 3:** Left atrial (LA) longitudinal stain values according to patent foramen ovale (PFO) and atrial sepal aneurysm (ASA) (values are mean ± SD).

	**PFO**			**PFO–ASA**			**Severe shunt**^**a**^		
	**Present**	**Absent**	***P***	**Present**	**Absent**		**Present**	**Absent**	***P***
	***N* = 21**	***N* = 30**		***N* = 10**	***N* = 41**	***P***	***N* = 12**	***N* = 37**	
LA global strain	40.7 ± 8.4	37.0 ± 8.3	0.108	39.9 ± 7.3	38.2 ± 8.8	0.602	41.7 ± 6.0	36.9 ± 8.7	0.054
LA active strain	17.0 ± 3.9	17.5 ± 5.4	0.592	17.7 ± 3.9	16.5 ± 5.0	0.434	16.8 ± 3.5	16.5 ± 5.1	0.625
LA passive strain	23.5 ± 8.1	20.3 ± 6.5	0.117	21.3 ± 6.4	21.7 ± 7.6	0.794	24.7 ± 8.2	20.2 ± 6.8	0.109

a *Missing data in two patients*.

**Figure 2:**
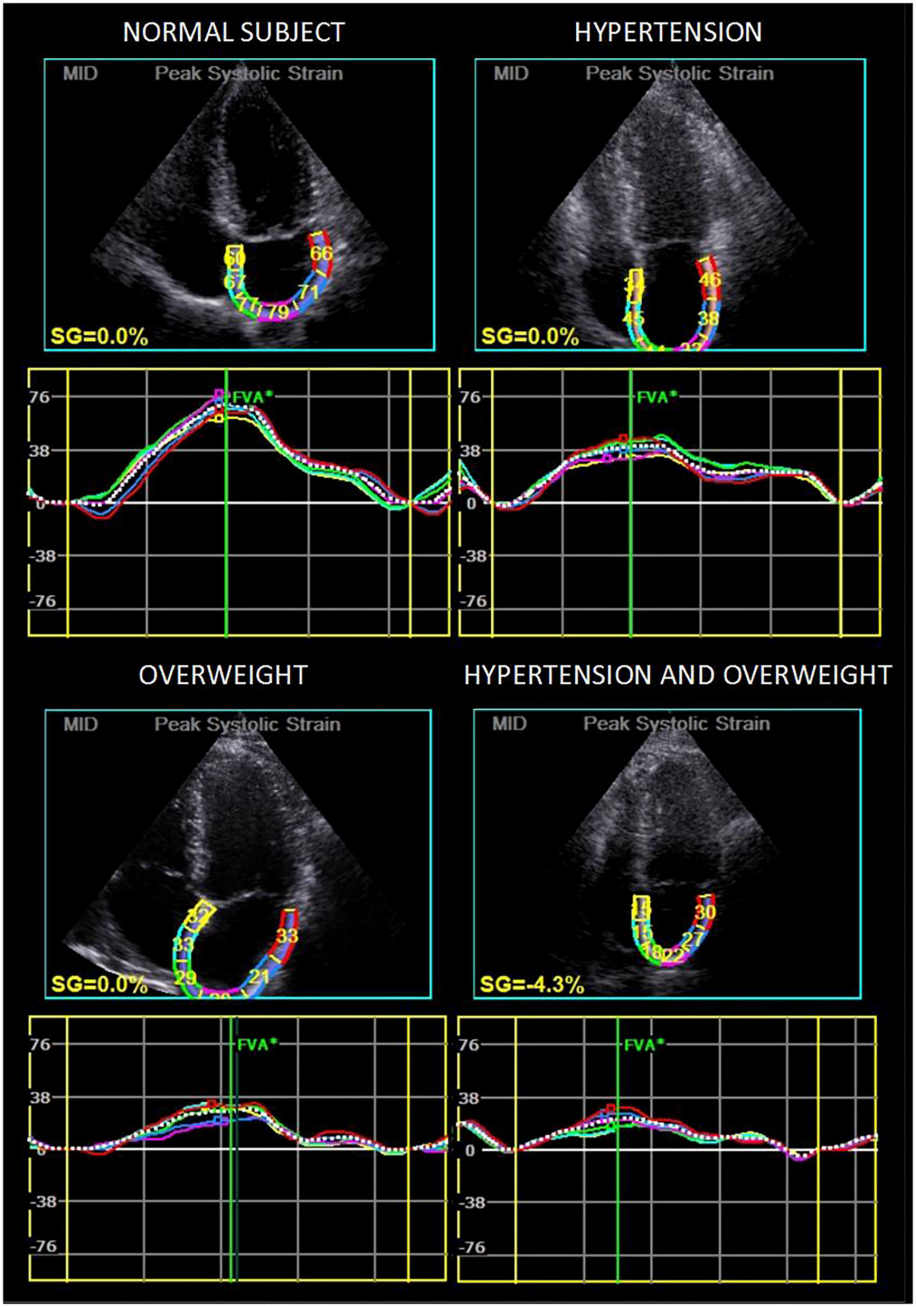
Strain curves in four illustrative patients showing longitudinal strain reduction in overweight and/or hypertensive patients.

A multivariable analysis using logistic regression showed associations of reduced LA global longitudinal strain with overweight (OR, 5.90; 95% CI, 1.45–23.99, *P* = 0.013), reduced LA active longitudinal strain with hypertension (OR, 5.95; 95% CI, 1.05–33.64, *P* = 0.049), and reduced LA passive longitudinal strain with overweight (OR, 6.87; 95% CI, 1.39–33.87, *P* = 0.018). Diabetes and dyslipidemia were not included in the models because there were too few patients with these risk factors and to limit the number of explanatory variables given the small sample size (Table 4 and Figure 2).

**Table 4:** Logistic regression analysis of associations between cardiovascular risk factors and patent foramen ovale (PFO) and reduced left atrial (LA) strain (reduced LA strain was defined as LA strain < median value).

	**LA global strain**		**LA active strain**		**LA passive strain**	
	**OR (95% CI)**	***P***	**OR (95% CI)**	***P***	**OR (95% CI)**	***P***
Age	1.01 (0.93–1.11)	0.660	1.00 (0.93–1.08)	0.884	1.10 (0.99–1.21)	0.060
Male	1.25 (0.30–5.11)	0.756	0.71 (0.19–2.63)	0.616	1.39 (0.33–5.85)	0.648
Overweight	5.90 (1.45–23.99)	0.013	0.84 (0.22–3.19)	0.802	6.87 (1.39–33.87)	0.018
Smoking	0.56 (0.13–2.36)	0.433	2.52 (0.66–9.66)	0.175	1.10 (0.23–5.16)	0.899
Hypertension	1.71 (0.29–9.95)	0.299	5.95 (1.05–33.64)	0.043	0.49 (0.07–3.17)	0.457
PFO	0.62 (0.14–2.74)	0.537	0.58 (0.15–2.17)	0.421	0.292 (0.06–1.33)	0.292

*LA, left atrial; OR, odds ratio*.

## Discussion

The present exploratory study using LA longitudinal strain measurement in young adults with CS showed associations of overweight with reduced LA reservoir function and reduced LA conduit function and of hypertension with reduced LA contractile function. In contrast, PFO, even in the presence of ASA or large right-to-left shunt, did not impact LA strain.

### LA Strain Measurement in Stroke Patients

Speckle tracking imaging, first developed for the analysis of LV deformation, has been validated by several studies for the assessment of LA function (12–14, 20). In addition to good reproducibility and angle independence, it detects a dysfunction at an earlier stage compared to classical parameters such as the size of LA, as functional changes precede morphological changes (9, 14). The measure of three different parameters is relevant as it corresponds to three components of LA function: reservoir, when LA fills with blood from pulmonary veins during systole; conduit, corresponding to the passage of blood into the ventricle during early diastole; and contractile, rising of LV stroke volume by LA contraction in late diastole. The use of LA longitudinal strain in this study was pertinent, as we expected a small degree of dysfunction, and LA longitudinal strain is easily obtainable in stroke patients, TTE being a part of the routine evaluation for cardio-embolic source.

Some previous studies used LA strain measurement in stroke patients. Most included older patients and focused on the link between LA dysfunction and cardiac diseases associated with a high risk of brain embolism such as LA thrombus or atrial fibrillation (5, 6, 21). Our study was not designed for analyzing this relationship, as only young patients with CS were included. We could find only one previous study of LA longitudinal strain in CS reporting reduced reservoir LA strain in patients compared to controls. Factors explaining this dysfunction were not explored but suggested to be linked to atherosclerosis risk factors (4).

### Association of Overweight and Hypertension With Impaired LA Strain

The finding of impaired LA function in the presence of hypertension and overweight is consistent with previous reports. The impact of hypertension and obesity on LA function are well-known in the general population, as they induce or contribute to atrial cardiomyopathy (22). These factors were associated with LA enlargement in earlier studies (23). More recent studies using the LA strain demonstrated impaired reservoir and conduit LA functions in patients with hypertension (24) in the absence of LA enlargement (7). A negative correlation between LA strain and body mass index was also reported (25). Our findings confirm the association of overweight with reduced reservoir and reduced conduit LA function and of hypertension with reduced contractile LA function in a selected population of young patients with CS.

### LA Function in Stroke Patients With PFO

PFO, especially PFO plus ASA or with large right-to-left shunt, is strongly associated with CS in young subjects (2, 17, 26). The prevalence of PFO and PFO plus ASA is higher in this population compared to the normal population and to patients with ischemic stroke of known etiology (27). The features of stroke associated with PFO are also different, both clinically, with younger patients less susceptible to have cardiovascular risk factors, and radiologically (26, 28). Several studies have now proven the benefit of transcutaneous PFO closure to prevent recurrent stroke in selected patients (29). However, the mechanisms of stroke associated with PFO remain unclear (30). Paradoxical embolism is often suspected but rarely proven. Other possible mechanisms include thrombus formation within the PFO or on the ASA surface and LA dysfunction. Two previous studies assessed LA function in stroke patients with PFO or ASA. Rigatelli et al. used conventional volumetric parameters in 98 stroke patients with PFO compared to 74 healthy subjects. They found significantly greater reservoir function and passive and active LA emptying, with significantly reduced conduit function and LA ejection fraction in patients with PFO compared to controls. Patients with PFO plus ASA had worse functional parameters than patients with isolated PFO (10). Na et al. compared 38 CS patients with isolated ASA to 38 age- and sex-matched healthy controls. The CS patients had significantly larger LA volume and lower active LA emptying fraction than the controls (11). In the present study of 51 CS patients using speckle tracking parameters, we found no evidence of impaired LA function in patients with PFO or PFO plus ASA compared to patients without. Among conventional LA function parameters, only LA total emptying volume was decreased in patients with PFO. It is possible that our negative findings were explained by a relatively small sample size. However, despite limited statistical power, we were able to confirm the association of LA dysfunction with hypertension and overweight. There are notable differences between the present study and the previous studies which may possibly explain the discrepant results. In the study by Rigatelli et al. the etiological workup of stroke was unspecified, and data on hypertension, diabetes, and overweight were not reported (10). Na et al. included only patients with isolated ASA (11). More importantly, stroke patients with PFO, or ASA were compared to healthy controls in both studies, whereas we compared CS patients with PFO to CS patients without PFO.

### Limitations

The present study has the general limitations of retrospective studies. Atrial fibrillation may have been overlooked in some patients, as we did not use long-duration recordings with implantable cardiac monitors. The measurement of the LA strain was performed with knowledge of the diagnosis of PFO in some patients. The large number of comparisons exposed to the risk of false positive results. In addition, the small sample size and the small number of patients with diabetes and hyperlipidemia did not allow these variables to be included in the multivariable analysis. However, the associations between overweight and hypertension and impaired LA function are consistent with the findings of previous studies. Finally, some of the negative results may have been due to insufficient statistical power. Therefore, our conclusions need to be confirmed on a larger sample.

## Conclusion

Impairment of LA longitudinal strain in young CS patients was not linked to PFO or PFO plus ASA but to overweight and hypertension. Further study is needed to confirm these findings in a larger number of patients.

## Data Availability Statement

All datasets generated for this study are included in the article/Supplementary Material.

## Ethics Statement

The studies involving human participants were reviewed and approved by CHU de Toulouse Institutional Review Board (internal reference RnlPH 2019-73). Written informed consent for participation was not required for this study in accordance with the national legislation and the institutional requirements.

## Author Contributions

JG, VL, and OL conceived the study. JG, CG, PF, and EC collected data. JG, VL, and OL analyzed and interpreted the data and drafted the manuscript. All authors contributed to the article and approved the submitted version.

## Conflict of Interest

The authors declare that the research was conducted in the absence of any commercial or financial relationships that could be construed as a potential conflict of interest.
